# Multiple Glacial Refugia and Complex Postglacial Dynamics of *Primula sikkimensis* (Primuaceae) in the Heterogeneous Qinghai‐Tibet Plateau

**DOI:** 10.1002/ece3.73024

**Published:** 2026-02-03

**Authors:** Hua‐Ying Sun, Yu‐Ting He, Zhi‐Hua Zeng, Yuan‐Mi Wu, Li Zhong, Qing‐Hong Feng, Hui‐Ying Gong, Xin Wang, Hong Wang, Zhi‐Kun Wu, Wei Zhou

**Affiliations:** ^1^ College of Chinese Materia Medica Yunnan University of Chinese Medicine Kunming China; ^2^ Germplasm Bank of Wild Species & Yunnan Key Laboratory of Crop Wild Relatives Omics, Kunming Institute of Botany, Chinese Academy of Sciences Kunming China; ^3^ Yongzhou Vocational Technical College Yongzhou China; ^4^ University of Chinese Academy of Sciences Beijing China; ^5^ Yunnan Provincial Key Laboratory of Forest Plant Cultivation and Utilization, Yunnan Academy of Forestry and Grassland Kunming China; ^6^ Research Institute of Tropical Forestry, Yunnan Academy of Forestry and Grassland Kunming China; ^7^ Department of Pharmacy Guizhou University of Traditional Chinese Medicine Guiyang China; ^8^ Key Laboratory of Phytochemistry and Natural Medicines, Kunming Institute of Botany, Chinese Academy of Sciences Kunming China

**Keywords:** Hengduan Mountains, Himalayas, lineage differentiation, phylogeography, *Primula*, Qinghai‐Tibet Plateau

## Abstract

The Qinghai‐Tibet Plateau (QTP) is a global biodiversity hotspot where Quaternary climatic oscillations profoundly shaped the evolution of endemic alpine flora. Understanding how genetic diversity and structure in these species responded to past climate change is crucial for deciphering regional evolutionary mechanisms. Using chloroplast and nuclear genome data of 958 samples from 48 populations, we evaluated the genetic diversity and population structure of *Primula sikkimensis*. We then investigated the lineage differentiation and dynamics of species by combining an Approximate Bayesian Computation procedure and species distribution modeling. Our study indicates that *P. sikkimensis* maintained separate glacial refugia in the Hengduan Mountains and eastern Himalayas during the Last Glacial Maximum (LGM). Our results suggest that postglacial range expansions onto the inner QTP plateau were accompanied by gene flow arising from both intraspecific secondary contact between previously isolated populations and interspecific hybridization events, which collectively enhanced genetic diversity and adaptive capacity in plateau populations. Our findings underscore the critical role of postglacial population dynamics and gene flow in shaping genetic diversity and adaptive potential of alpine endemics like *P. sikkimensis* , highlighting evolutionary responses to Quaternary climate change on the QTP.

## Graphical abstract

Primula sikkimensis persisted in distinct glacial refugia within the Hengduan Mountains and eastern Himalayas during the Last Glacial Maximum. Subsequent postglacial range expansions onto the Qinghai–Tibet Plateau were accompanied by secondary contact and interspecific hybridization, collectively enhancing genetic diversity and adaptive capacity. These results demonstrate the pivotal role of refugial persistence and postglacial gene flow in shaping evolutionary responses of alpine endemics to Quaternary climatic oscillations.

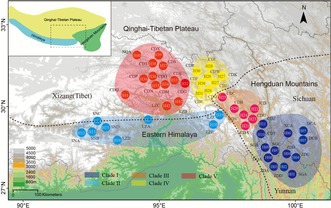

## 1 Introduction

**FIGURE 1:**
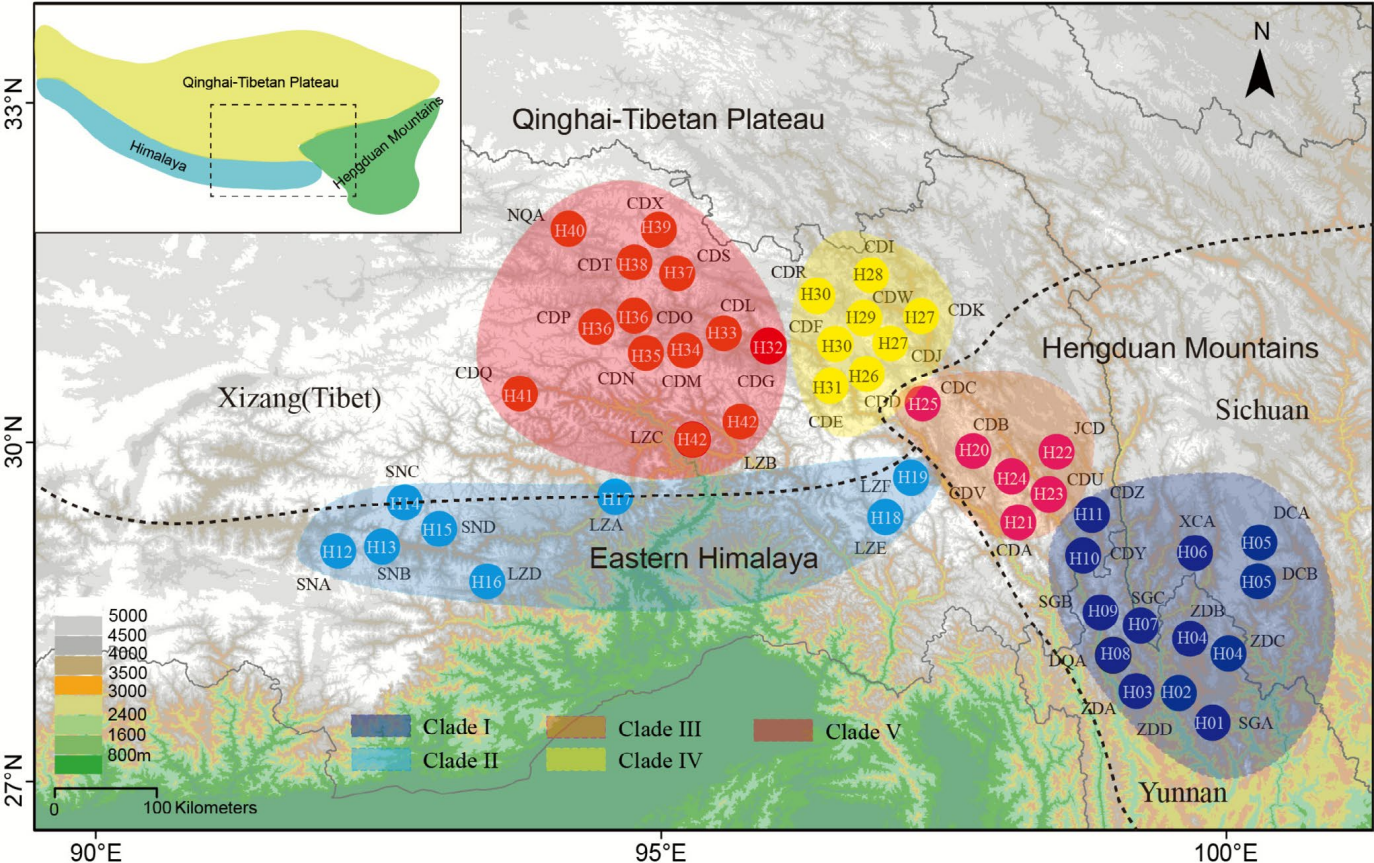
Geographic distribution of the 48 *Primula sikkimensis* populations investigated in this study. Each population (localities listed in Table S1) is distinguished by a letter‐ coded circle. Circle color indicates the lineage to which the population belongs: In purple for clade I, blue for clade II, orange for clade III, yellow for clade IV, and red for clade V. Internal codes within circles represent the distribution of chloroplast (cp) haplotypes within each population. Dotted lines delineate the main areas of the Qinghai‐Tibetan Plateau, Eastern Himalaya, and Hengduan Mountains.

The Qinghai‐Tibet Plateau (QTP) *sensu lato* (*sl*) comprises the QTP *sensu stricto* (*ss*, or the platform), the Himalaya, and the Hengduan Mountains (Figure 1), represents one of the most biologically diverse and geologically dynamic regions on Earth (Wen et al. 2014; Mao et al. 2021; Wu et al. 2022). This area, known as the “Roof of the World”, has undergone dramatic tectonic uplift and climatic changes since the early Miocene (Guo et al. 2002, 2008; Spicer et al. 2003), making it characterized by extreme environmental heterogeneity, ranging from high‐altitude alpine meadows to deep river valleys. This topographical complexity has created numerous microhabitats and acted as both a barrier and a corridor for plant dispersal which have played pivotal roles in driving speciation and diversification (Wen et al. 2014; Sun et al. 2017). At present, the QTP *sl* is estimated to home to at least 12,000 species of vascular plants in about 1500 genera, and more than 20% of the total species are endemic to the region (Wu and Wu 1996; Sun et al. 2017; Gao et al. 2025). It serves as a center of diversity for numerous species‐rich genera. Examples include *Rhododendron* (Ericaceae), *Pedicularis* (Orobanchaceae), *Corydalis* (Papaveraceae), *Gentiana* (Gentianaceae), *Kobresia* (Cyperaceae), *Stipa* (Poaceae), *Primula* (Primulaceae), and *Saussurea* (Asteraceae), all harboring high numbers of endemic species (Wu and Wu 1996; Sun et al. 2017).

The geological events coupled with Pleistocene glacial cycles have profoundly influenced the genetic structure and distribution of both plant and animal populations in the QTP *sl* (Yang et al. 2009; Liu et al. 2014). Studies on plant phylogeography in this region have revealed several recurring patterns (reviewed in Qiu et al. 2011; Liu et al. 2012). For instance, many plant species exhibit strong genetic differentiation between populations, often corresponding to major mountain ranges or river systems (e.g., Zhang et al. 2005; Wang et al. 2009; Yang et al. 2012; Liu et al. 2025). This suggests that geographical barriers have limited gene flow and promoted allopatric speciation. Additionally, glacial refugia, particularly in the southeastern QTP *ss* and the Himalaya‐Hengduan Mountains (HHM), have been identified as critical areas where plant species survived during glacial periods (e.g., Wang et al. 2009, 2019; Liu et al. 2013; Yang and Zhou 2023). Post‐glacial recolonization from these refugia has led to complex patterns of genetic diversity, with some species showing evidence of range expansion, secondary contact between previously isolated lineages, and hybrid speciation (Wu et al. 2022). As climate change and human activities continue to impact this fragile ecosystem, understanding the phylogeographic patterns of its flora becomes increasingly important for conservation and management efforts (Muellner‐Riehl 2019).

Recent advances in molecular techniques, particularly next‐generation sequencing (NGS), have revolutionized the resolution and scope of plant phylogeographic studies in the QTP *sl* (Muellner‐Riehl 2019; Zhang et al. 2020; Wu et al. 2022). These powerful tools enable detailed reconstruction of population histories, uncovering complex evolutionary processes that were previously intractable. These studies have not only improved our understanding of the evolutionary history of individual species but also provided broader insights into the mechanisms driving biodiversity assembly in mountainous regions (Chen et al. 2019; Li et al. 2021; Ma et al. 2021). However, significant challenges persist, including the need for more extensive sampling across taxa and the integration of ecological and climatic data to better understand the interplay between genetic diversity and environmental factors.

The genus *Primula* (Primulaceae), prominently featured among the species‐rich genera endemic to the QTP *sl* and a quintessential alpine component of the HHM (Hu and Kelso 1996; Richards 2003), serves as a prime exemplar of the region's diversification dynamics. Its center of diversity and endemism is firmly rooted within this biodiversity hotspot, where complex topography, pronounced climatic heterogeneity, and prolonged glacial–interglacial cycles have sculpted its evolutionary trajectory (Xie et al. 2012; Yan et al. 2012; Ren et al. 2015, 2017, 2018). However, phylogeographic investigations within *Primula* remain heavily skewed toward European species (e.g., *P. vulgaris* and *P. veris* ), where post‐glacial recolonization routes and genetic bottlenecks are comparatively well characterized (Volkova et al. 2020; Mora‐Carrera et al. 2024; Triest et al. 2024). In contrast, the hyperdiverse Asian lineages, particularly in alpine regions, remain understudied though they exhibit high endemism and unique adaptive radiations. Current studies emphasized the role of mating system divergence in shaping species distributions and genetic structure (Zhong et al. 2019; Wang et al. 2021; Yuan et al. 2023; Zeng et al. 2024; Sun et al. 2025). Most *Primula* species possess a distylous, self‐incompatible mating system, characterized by populations containing both long‐styled and short‐styled floral morphs that promote pollinator‐mediated outcrossing (Richards 2003). Evolutionary shifts from this obligate outcrossing to high selfing homostyly in some species have been linked to expansions into marginal habitats, whereas the persistence of distyly in core populations enhances genetic diversity and resilience (Yuan et al. 2017; Zhou et al. 2017; Zhang et al. 2021). Owing to its wide yet patchy distribution across mountain ranges and altitudinal gradients of the QTP *sl*, along with its predominant outcrossing system and limited seed dispersal, the genetic structure of *Primula* is especially sensitive to historical population isolation and expansion events. Consequently, phylogeographic studies of *Primula* in the QTP *sl* region could offer unique insights into how Quaternary climate change, mating system evolution, and mountain geodynamics have jointly shaped biodiversity patterns.

In this study, we focus on *Primula sikkimensis* (Primulaceae), one of the most widely distributed alpine plant species in the HHM and Tibetan Plateau (Hu and Kelso 1996; Richards 2003). *Primula sikkimensis* is a diploid (2*n* = 22) perennial herb that commonly occurs in moist meadows, on open slopes, and beside hill streams at elevations ranging from 3300 to 5000 m (Hu and Kelso 1996). As recorded, *P. sikkimensis* exhibits distyly (Richards 2003), with populations comprising long‐styled and short‐styled floral morphs enabling obligate outcrossing mediated by pollinators. Previous phylogeographic analyses indicated that *P. sikkimensis* presented high genetic diversity and population differentiation (*G*_ST_ = 0.697) and limited gene flow mediated by seed and pollen are likely to have played important roles in its intraspecific divergence (Wang et al. 2008). This species hence represents an ideal system to evaluate the effects of past climatic changes on a species' evolutionary history in the QTP *sl*. Here, we employ an integrative approach that combines whole chloroplast genome and nuclear markers with niche modeling to elucidate the divergence and demographic history of *P. sikkimensis* . Our study aims to: (1) identify the phylogeographic patterns of this species and the factors that may have triggered its intraspecific divergence; (2) contrast the potential differences in the resolution and patterns of lineage differentiation inferred from plastid and nuclear markers; and (3) reconstruct the demographic history of *P. sikkimensis* while evaluating the potential effects of Quaternary climatic changes using the species distribution models.

## 2 Materials and Methods

### 2.1 Population Sampling and DNA Extraction

We collected a total 958 individuals from 48 populations of *P. sikkimensis*. These materials were sampled from Yunnan, Sichuan, and Tibet provinces and covered most of the distribution areas of this species (Figure 1 and Table S1). For each population, we randomly sampled 18–20 individuals, ensuring that the sampling locations were at least separated 10 m apart. Fresh leaves were collected and dried immediately in silica gel for DNA extraction. Voucher specimens were deposited in the herbarium of Kunming institute of Botany (KUN).

To investigate the phylogeography of *P. sikkimensis* , we used 48 samples (one sample per population) to obtain chloroplast genome assemblies and 958 samples (15–20 plants per population) for nuclear microsatellite genotyping. Total genomic DNA was extracted from 20 mg silica‐dried leaves using a modified cetyl trimethyl ammonium bromid (CTAB) protocol (Doyle 1991). Quantification of DNA was conducted using agarose gel electrophoresis (1%), and we determined concentrations using a SmartSpec Plus Spectrophotometer (Bio‐Rad, Hercules, CA, USA). The qualified DNA samples were diluted to a concentration of 10 ng/mL and preserved at −20°C for subsequent use.

### 2.2 Phylogeographic Inference Using Chloroplast Genome Data

We selected 48 samples each from a population for sequencing and whole chloroplast genome assembly. The purified genomic DNA was then sheared into *c*. 500 bp fragments to construct a paired‐end (PE) library according to the Nextera XT sample preparation procedures (Illumina, San Diego, CA, USA). We generated the PE reads of 300 bp using an Illumina HiSeq 2500 sequencer at the Beijing Genomic Institute (Shenzhen, Guangdong, China) or Illumina MiSeq instrument at the Laboratory of Molecular Biology of Germplasm Bank of Wild Species (Kunming Institute of Botany, CAS). We obtained *c*. 3 Gb of sequence data for each of 48 individuals, with one sample obtained from each population.

We performed sequence quality trimming using the NGS QC TOOL Kit (Patel and Jain 2012) with default parameters. We obtained de novo assembly of high‐quality short reads using Getorganelle v.1.7.7 (Jin et al. 2020) with a k‐mer of 121. Reads were mapped back to assemblies using BOWTIE v.2.2.3 (Langmead and Salzberg 2012) to verify the assembly quality. We annotated the complete plastomes using PGA v.2.0 (Qu et al. 2019), which implements BLAST and a fully annotated plastome database to predict protein coding genes, transfer RNA (tRNA), and ribosome RNA (rRNA). We confirmed the boundaries of intron/exon manually by aligning their orthologs using other published information from congeneric species.

We generated two data sets for phylogeographic analyses; a whole chloroplast genome alignment performed using MAFFT v.7.487 (Katoh and Standley 2013) and a concatenated data set comprised of coding sequence (CDS) of the 77 protein‐coding genes in the plastome. We retrieved haplotypes using the program DnaSP v.6.0 (Rozas et al. 2017). We investigated phylogenetic relationships among haplotypes using maximum likelihood (ML) and Bayesian inference (BI) methods, with *P. alpicola* and *P. tibetica* as outgroups. We used the program MODELTEST‐NG v.0.1.6 (Darriba et al. 2020) with the Akaike Information Criterion (AIC) to determine the most suitable base substitution model. We performed ML analysis under the program RAxML v.8.2.12 (Stamatakis 2014) with 1000 pseudoreplicates of rapid bootstrap and used MRBAYES v.3.2.7 (Ronquist and Huelsenbeck 2003) to infer the optimal tree topology and calculate the posterior probability for the dataset. Haplotype networks were constructed using the software PopART v.1.7 (Leigh and Bryant 2015). To assess variation in genetic diversity among the inferred lineages, we estimated population statistics of pairwise nucleotide diversity (*π*), Watterson's theta (*θ*_W_), and Tajima's *D* for both synonymous and nonsynonymous sites of chloroplast genome using the program DnaSP v.6.0 (Rozas et al. 2017).

### 2.3 Phylogeographic Inference Using Microsatellite Genotype Data

Using 10 microsatellite primer pairs (S12, S18, PK74, PK99, PK78, PK76, PK77, PK73, PK92, PK75) developed for *P. sikkimensis* , we scored genotypes in 958 individuals (Table S1). We performed PCR amplification using the following protocol: 25 μL total reaction volume containing 10 μL of Master Mix (Tiangen Biotech, Beijing, China; including 3 mM MgCl_2_, 100 mM KCl, 0.5 mM of each dNTP, 20 mM Tris–HCl, pH 8.3 and 0.1 units Taq polymerase), 0.3 μL of each primer (the 5′ side of the forward primers were labeled with fluorescent dye: HEX, TAM or 6‐FAM), 8.4 μL of deionized water and 20 ng of genomic DNA. We conducted PCR amplifications on a thermocycler (Perkin‐Elmer, Foster City, CA, USA) under the following conditions: 95°C for 3 min, followed by 30 cycles at 95°C for 30 s for denaturation, 1 min for annealing, 72°C for 1.5 min for extension, and a final extension step at 72°C for 10 min. We checked PCR products on 1% agarose gels stained with ethidium bromide. All PCR products were separated and visualized using an ABI 3730 XL automated sequencer (Applied Biosystems, Foster City, CA, USA). We first determined allele sizes using GENEMAPPER 3.7 software with GeneScan‐500 ROX as an internal‐lane size standard (Applied Biosystems) and then rechecked the data manually to reduce scoring errors. Five randomly selected PCR products for each locus were purified and sequenced in both directions to examine the character of the repeat motif. Sequencing primers were identical with those used in the PCR, and were conducted in forward and reverse reactions individually.

In the analysis, the raw data matrix generated by microsatellite markers was examined using the GenAlEx v.6.5 (Peakall and Smouse 2006). We estimated allele frequencies at microsatellite loci, as well as the mean number of alleles per locus (*N*_A_), observed and expected heterozygosity (*H*_O_, *H*_E_), and effective number of alleles (*N*_E_) using the program FSTAT v.2.9.3 (Goudet 1995).

To assess the phylogeographic relationships among populations, we used several approaches to investigate the patterns of differentiation among populations. On the basis of the original genotype data, we calculated a Euclidean distance matrix among all populations. The genetic relationships were then visualized using principal coordinate analysis (PCoA) implemented in the program MVSP v.3.1 (Kovach 1999) and neighbor‐net analysis performed with the R package phangorn (Bryant and Huson 2023). We calculated Nei's (1987) unbiased genetic distance among all possible pairs of populations from allele frequencies estimated in the program microsatellite analyzer (MSA) (Dieringer and Schl€otterer 2003). We then constructed a consensus neighbor‐joining (NJ) tree based on pairwise estimates of genetic distance, using 1 × 10^3^ bootstrap trees and random input order in PHYLIP v.3.63 (Felsenstein 2005). Second, we used the program STRUCTURE v.2.3.4 (Pritchard et al. 2000) to infer the number of genetic clusters (*K*) in our entire dataset under the “no admixture” and “uncorrelated allele frequencies” model. Clusters were set from 2 to 8, and 10 independent runs were performed at 1 × 10^6^ Markov Chain Monte Carlo simulations after a burn‐in of 5 × 10^5^ iterations. We estimated the most likely number of clusters based on the log probability of the data between successive *K* values (Evanno et al. 2005). Finally, a hierarchical analysis of molecular variance (AMOVA) was performed in Arlequin v.3.5 (Excoffer et al. 2005) with 1 × 10^4^ permutations to estimate the level of variation among and within groups. The grouping of populations was based on the optimal clustering suggested by STRUCTURE.

We deciphered the historical demography of *P. sikkimensis* by estimating divergence time, admixture and changes in population size among different population groups using the program DIYABC v.2.1.0 (Cornuet et al. 2008). We pooled the population samples into five ‘groups’ (i.e., clades I–V) based on the main characteristics of lineage clustering that were captured by the NJ tree and STRUCTURE analysis. Six competing scenarios of population history were summarized (Figure S1) and simulated under an approximate Bayesian computation (ABC) procedure (Beaumont et al. 2002). We gave each scenario a uniform prior probability and selected the summary statistics to generate a reference table containing 4 × 10^5^ repeats for each model. We selected the 1 × 10^5^ (20%) simulated datasets closest to the observed data to estimate the relative posterior probabilities and 95% confidence intervals (CI) for each model via logistic and posterior distribution of demographic parameters according to the most likely scenario (Cornuet et al. 2008). The time parameters are estimated in generations and converted into years by multiplying generation time, which was set to 1 year for *P. sikkimensis* according to field observations and other studies on related species of *Primula* (Yan et al. 2012; Ren et al. 2017). According to the inference of lineages, we estimated the gene flow (2 *N*m) for pairwise clades using a coalescent‐based Bayesian inference implemented in the MIGRATE‐N v.3.6.11 software (Beerli and Palczewski 2010).

### 2.4 Species Distribution Models (SDMs)

We generated the species distribution models (SDMs; Guisan and Zimmermann 2000) for *P. sikkimensis* using the MAXENT v.3.2 in the R package biomod2 (Thuiller et al. 2009). A total of 288 species occurrences obtained directly from field observations (48) and historical records (240) from the Global Biodiversity Information Facility (GBIF; http://data.gbif.org) and the National Plant Specimen Resource Center (NPSRC; https://www.cvh.ac.cn) were used as presence data to calibrate the models. We used the 19 BIOCLIM variables of the WorldClim dataset (http://www.worldclim.org; Hijmans et al. 2005) as environmental predictors. To avoid multicollinearity, we ran a Pearson correlation analysis to eliminate one of the variables in each pair with a correlation value higher than 0.8 (Dormann et al. 2013). A set of nine variables was finally used to carry out the SDM. The consensus model was converted to a binary model (presence/absence) and then projected onto different climatic periods using the data available in the WorldClim: the Last Interglacial (LIG; 0.12–0.14 Ma), the Last Glacial Maximum (LGM; 0.022 Ma), and the mid‐Holocene (MH; 0.006 Ma). We employed three different general circulation models including CCSM4 (Community Climate System Model, version 4), MIROC‐ESM (Model for Interdisciplinary Research on Climate, Earth System Model), and MPI‐ESM‐P (Max Planck Institute Earth System Model, Paleoclimate version) for the MH and LGM.

## 3 Results

### 3.1 Lineage Differentiation Inference from Chloroplast Genome

We obtained 48 newly generated complete plastid genomes of *P. sikkimensis* which displayed a consistent structure, gene content and gene order. The whole plastid dataset had an aligned length of 155,547 bp with 1293 parsimony informative characters (PICs, 0.83%). By removing the intergenic regions and a few introns, the CDS dataset had an aligned length of 66,231 nucleotides with a reduced number of PICs of 445 (0.67%). The whole plastome dataset exhibited considerable polymorphism and comprised a total of 42 haplotypes (H1‐H42). The geographical distribution of haplotype is illustrated in Figure 1, most of *P. sikkimensis* populations (87.5%) contained a unique haplotype and six haplotypes (H4, H5, H27, H30, H36, and H39) were shared by two populations (Figure 1).

**FIGURE 2:**
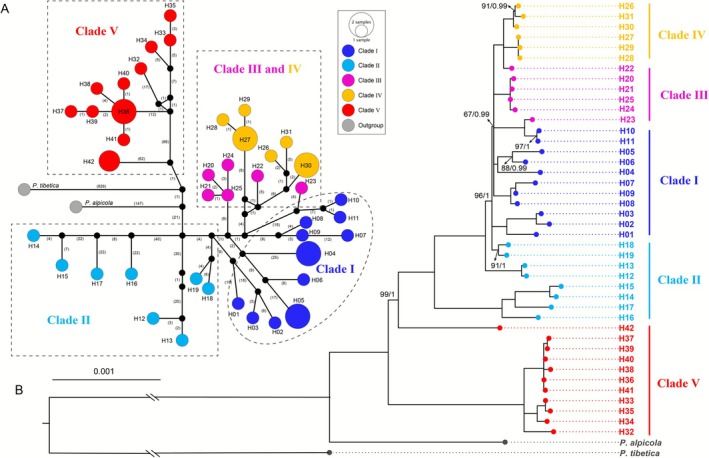
Haplotype phylogenetic framework of population samples from *Primula sikkimensis*. (A) Haplotype network constructed from concatenated dataset of coding sequence (CDS) from the 77 protein‐coding genes in the chloroplast genome. (B) Haplotype phylogenetic framework constructed using the chloroplast genome dataset. The topology is supported by both Maximum Likelihood (ML) and Bayesian Inference (BI) analyses. ML bootstrap values (BS < 100) and Bayesian posterior probabilities (PP < 1.0) are indicated at nodes. Purple, blue, orange, yellow, and red circles represent haplotypes of clade I, clade II, clade III, clade IV, and clade V, respectively. The distribution of corresponding haplotypes is indicated in Figure 1.

The haplotype tree obtained from ML and Bayesian methods was largely congruent in topology (Figure 2B), and the populations of *P. sikkimensis* were strongly supported as monophyletic (ML bootstrap value 100%; Bayesian posterior 1.00). All haplotypes were clustered into two major lineages with strong nodal supports, one super‐lineage including haplotypes H1‐H34 which was further divided into four clades (clades I–IV, see results of next Section 3) and the other distinct lineage (clade V) included remaining haplotypes (H35‐H42). The haplotype network (Figure 2A) largely confirmed the pattern revealed by tree topology, with the west QTP *ss* originated haplotypes (H35‐H42) forming a relatively separate group and connected to the eastern Himalayan haplotypes (H12‐H19). However, the eastern QTP *ss* haplotypes (H26‐H31) were derived from the haplotypes (H20‐H25) distributed in the northern marginal Hengduan Mountains region.

### 3.2 Lineage Differentiation Inference From Nuclear Microsatellite Markers

**FIGURE 3:**
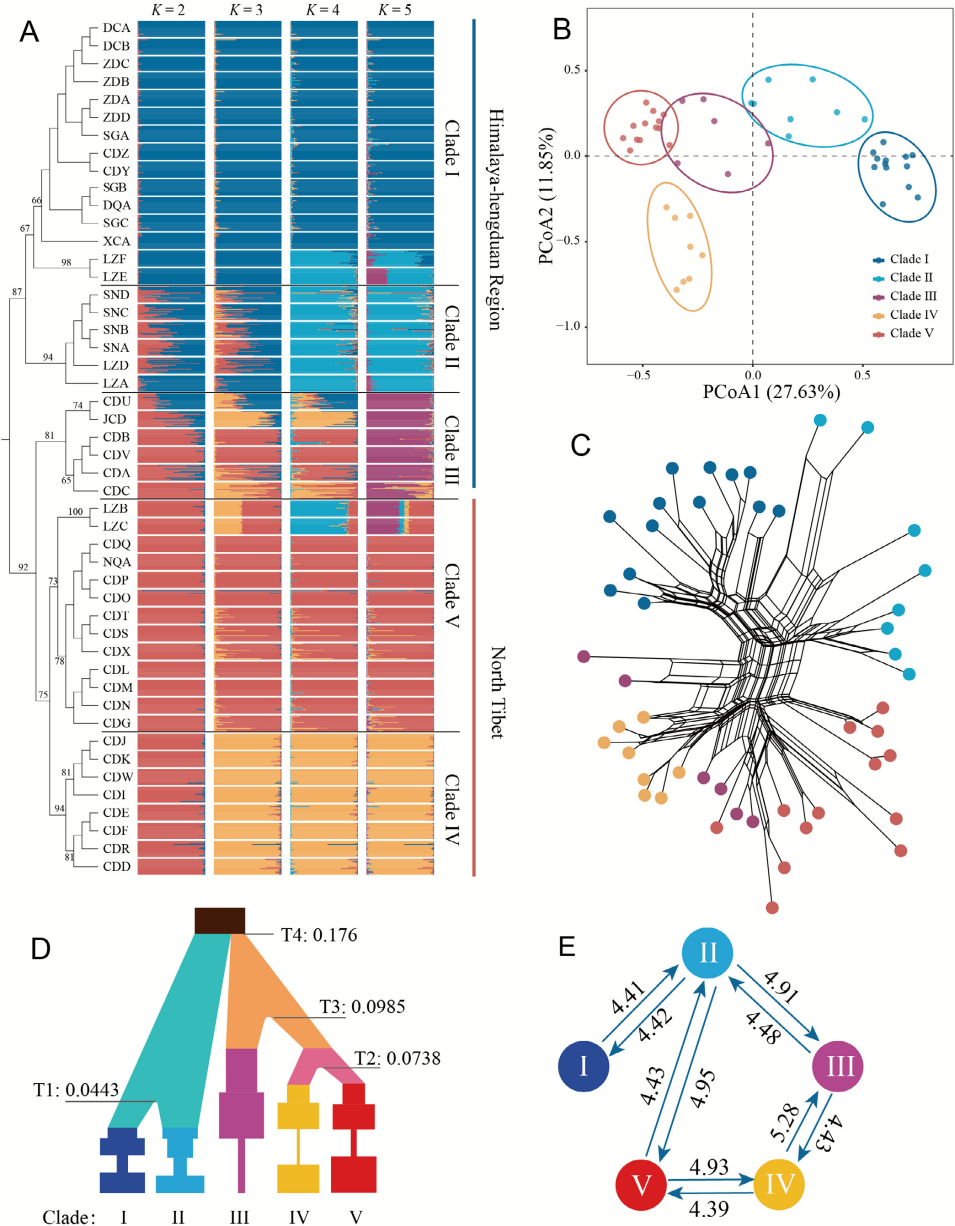
Population genetic structure, lineage divergence and gene flow of population samples from *Primula sikkimensis*. (A) Population cluster analysis (at *K* = 2–5) based on microsatellite genotypes of 958 individuals from 48 populations. Population codes and distributions are provided in Table S1 and Figure 1. (B) Principal coordinates analysis (PCoA) of the microsatellite genotypes of 48 populations of *P. sikkimensis* . (C) Neighbor‐net for the observed distances of microsatellite genotypes between 48 populations of *P. sikkimensis* . (D) Diagram of the best‐supported demographic model estimated by DIYABC (Model 4 in Figure S3). Each block represents a current or ancestral population, and the top block represents the common ancestor of five clades. Divergence times are indicated in Ma; changes of population size are indicated by the width of the blocks. The point estimates and 95% confidence intervals of all demographic parameters are presented in Table S3. (E) Estimated gene flow (2*Nm*) between clades, with arrows and numbers indicating the direction and strength of gene flow.

The population‐based NJ tree revealed two distinct clusters, i.e., HHM and QTP *ss*, with the genetic relationships among the populations reflecting their geographical origins (Figures 1, 3A). Populations from the HHM are divided into three major clades: clades I and III, which are primarily located in the core Hengduan Mountains region, and clade II, which encompasses the eastern Himalaya regions that extend from west to east and contact the Hengduan Mountains region (Figure 1). Two sub‐clusters were clearly present among the QTP *ss* populations: clades IV and V (Figure 1). This suggests significant genetic differentiation among populations from different geographical areas, likely due to historical isolation and local adaptation.

The STRUCTURE analysis recovered a local peak of delta *K* values at *K* = 5 (Figure S2), which further supports the genetic differentiation observed in the NJ tree. The results show that most populations have a predominant genetic component, but some exhibit admixture, indicating gene flow between populations or shared ancestral genetic backgrounds (Figure 3A). For example, the two populations, LZE and LZF, in clade I showed similarity to clade II when *K* was increased to 4 and 5. Similarly, the populations LZB and LZC in clade V displayed components of clade II and clade III when *K* was set at 4 and 5, respectively. Overall, the combined results from the NJ tree and STRUCTURE analysis highlight the complex genetic structure of *P. sikkimensis* populations, shaped by both geographical isolation and historical gene flow.

Two‐dimensional PCoA of the microsatellite phenotypes (Figure 3B) separates the clades I, II, III, and V along the first axis, with PCoA1 explaining 27.63% of the total variation. The populations of clade IV were separated from the remaining populations along the second axis (11.85%). The results of the neighbor‐net analysis generally support the conclusion of five lineage clades, with two exceptions (Figure 3C). First, the CDG population of clade IV is nested within clade V, while two populations from clade V show closer affinities to clade IV. These populations with unstable positions are all located in the geographical contact zones between the lineages, indicating the presence of substantial gene flow among them. Collectively, both chloroplast and nuclear markers support the division of this species into five distinct lineages. The minor discrepancies observed between the two sets of markers are primarily due to the fact that chloroplast DNA is maternally inherited while nuclear markers are biparentally inherited.

Significant genetic differentiation was evident at both the population and clade levels. Analysis of nuclear data revealed that the amount of the genetic variance (37.2%) was observed between populations. Specifically, hierarchical AMOVA demonstrated high differentiation between populations within clades (*F*_SC_ = 0.233, *p* < 0.01) and substantial differentiation between clades (*F*_CT_ = 0.182, *p* < 0.01). Pairwise *F*_ST_ analyses based on microsatellite markers consistently indicated significant genetic differentiation between clades, with values ranging from 0.129 to 0.251 (mean = 0.182). In addition, pairwise estimates of *F*_ST_ based on the chloroplast genome revealed even higher genetic differentiation, with a range from 0.105 to 0.760 (mean = 0.428).

### 3.3 Lineage Differentiation History Simulated by ABC Modeling

Approximate Bayesian Computation modeling of the demographic history of *P. sikkimensis* indicated that the posterior probabilities for evolutionary model 4 were 0.886 (95% CI: 0.855–0.918), significantly higher than the other scenarios (model 1: 0.002, model 2: 0.035, model 3: < 0.001, model 5: 0.065, model 6: < 0.001). Model 4 depicted an evolutionary history in which clade I and clade II diverged from an ancestral lineage, while the other three clades (III, IV, and V) formed a closer lineage that was sequentially derived from a common ancestor (Figure 3C). The estimated posterior distribution parameters for each model are presented in Table S2.

We estimated the divergence times and the population sizes (Table S3), along with the timing and extent of their changes for the five clades in the best‐fitting model (model 4). Our ABC modeling suggested that *P. sikkimensis* diverged first *c*. 0.176 Ma (95% CI: 0.141–0.198), and clade I and II originated from a common ancestor lineage *c*. 0.0443 Ma (95% CI: 0.0097–0.0812). Clade III originated in *c*. 0.098 Ma (95% CI: 0.049–0.143), and clades IV and V was more recently *c*. 0.073 Ma (95% CI: 0.0039–0.0974). After the divergences, all clades experienced a period of founder events and started to expand their population sizes except clade III, which consistently maintained a small population size (2.26 × 10^5^) over time (Figure 3C). The estimated posterior distribution parameters for each clade are presented in Table S3.

### 3.4 Genetic Diversity Patterns and Gene Flow Among Lineages

Genetic diversity indices are listed in Tables 1 and 2 for chloroplast genome and nuclear microsatellite variations, respectively. For the chloroplast coding regions, the nucleotide diversity for both synonymous ( *π*_S_ ) and nonsynonymous ( *π*_N_ ) sites were much higher for clade I ( *π*_S_ = 0.163%; *π*_N_ = 0.04%), clade II ( *π*_N_ = 0.274%; *π*_S_ = 0.085%) and clade V ( *π*_S_ = 0.185%; *π*_N_ = 0.060%) than for those in clade III ( *π*_S_ = 0.056%; *π*_N_ = 0.020%) and IV (*π*_S_ = 0.034%; *π*_N_ = 0.014%). These general trends of diversity variation were also refracted by the measurement of Watterson's theta ( *θ*_W_ ) (Table 1). The ratio of nonsynonymous to synonymous nucleotide diversity ( *π*_N_ : *π*_S_ Or *θ*_N_ : *θ*_S_ ) was lower in Himalaya (Clade II: 0.310, 0.325) and core Hengduan Mountains clade (clade I: 0.245, 0.257), and sequentially increased in clade III and clade IV along the route of expected population expansion (Table 1). However, the *π*_N_ : *π*_S_ or *θ*_N_ : *θ*_S_ of clade V (0.324, 0.325) were not the lowest among clades as the frontier populations predicted by the lineage differentiation model.

**TABLE 1:** Summary of chloroplast genome diversity for the studied five lineages of the *Primula sikkimensis*. Nucleotide diversity (*π*), Watterson's theta (*θ*_W_) and test for neutrality (Tajima's *D* based on *π* and *θ*_W_) for each lineage are given.

Lineage	*H*	Site class	*N*_sites_	*S*	*π* (10^−2^)	*θ*_W_ (10^−2^)	*π*_N_:*π*_S_	*θ*_N_:*θ*_S_	Tajima's *D*
Clade I	11	Synonymous	15,199	90	0.163	0.191	0.245	0.257	−0.659
		Nonsynonymous	50,818	78	0.040	0.049			−0.880
Clade II	8	Synonymous	15,165	99	0.274	0.240	0.310	0.325	0.580
		Nonsynonymous	50,690	102	0.085	0.078			0.410
Clade III	6	Synonymous	15,215	20	0.056	0.058	0.357	0.431	−0.209
		Nonsynonymous	15,161	29	0.020	0.025			−1.313
Clade IV	6	Synonymous	15,215	11	0.034	0.028	0.412	0.429	1.102
		Nonsynonymous	50,865	16	0.014	0.012			0.631
Clade V	11	Synonymous	15,270	99	0.185	0.209	0.324	0.325	−0.582
		Nonsynonymous	51,014	107	0.060	0.068			−0.582

*Note:* *θ*_w_, Watterson's theta; *π*, nucleotide diversity; *H*, the number of haplotypes; *N*_sites_, number of sites; *S*, number of polymorphisms.

**TABLE 2:** Summary of 10 microsatellite loci genotyped on 958individuals of *Primula sikkimensis*. Number of individual (*N*), number of alleles per locus (*N*_A_), number of effective alleles per locus (*N*_E_), Shannon's index (*I*), observed and expected heterozygosities (*H*_O_ and *H*_E_), and inbreeding coefficient (*F*_IS_) at each locus.

Locus	*N*	*N*_A_	*N*_E_	*I*	*H*_O_	*H*_E_	*F*_*IS*_	Size range (bp)
S12	955	23	9.018	2.540	0.468	0.889	0.474	136–187
S18	944	22	2.070	1.487	0.460	0.517	0.110	150–207
PK74	950	27	16.422	2.954	0.763	0.939	0.187	184–262
PK99	935	23	7.149	2.256	0.733	0.860	0.148	156–237
PK78	928	12	2.764	1.376	0.179	0.638	0.720	297–336
PK76	926	20	5.758	2.046	0.463	0.826	0.439	227–284
PK77	930	15	5.941	1.955	0.417	0.832	0.498	130–181
PK73	932	15	3.229	1.677	0.319	0.690	0.538	252–294
PK92	950	13	5.790	1.971	0.547	0.827	0.338	262–298
PK75	947	10	4.099	1.610	0.439	0.756	0.419	156–186

The nuclear microsatellite loci we investigated in *P. sikkimensis* were highly polymorphic (Table 3), with an average of 18 alleles per locus. Population genetic parameters based on 10 loci for each population are presented in Table S4. For the nuclear microsatellite data, we revealed similar trends to chloroplast genome in the variation of genetic diversity among clades of *P. sikkimensis* (Table 3). Overall, the genetic diversity indices, including the values of *N*_A_, *N*_E_, *I*, *H*_O_ and *H*_E_, were significantly higher in clades I, II, and V compared with clades III and IV. Consistent with the prediction of a distylous species, the level of inbreeding was close to zero in *P. sikkimensis*, and clades III (0.109 ± 0.083) and IV (0.086 ± 0.116) presented slightly higher values of inbreeding coefficient (*F*_IS_) than those of other clades.

**TABLE 3:** Parameters of the genetic diversity estimated from microsatellite genotypes for each lineage of *Primula sikkimensis*. Population number (*N*_Pop_), individual number (*N*_Ind_), the mean number of alleles per locus (*N*_A_), observed and expected heterozygosity (*H*_O_, *H*_E_), effective number of alleles (*N*_E_), and fixation index (*F*) for each lineage are given.

Lineage	*N*_Pop_	*N*_Ind_	*N*_A_ ± SE	*N*_E_ ± SE	*I* ± SE	*H*_O_ ± SE	*H*_E_ ± SE	*F* ± SE
Clade I	13	258	5.838 ± 0.587	3.169 ± 0.216	1.234 ± 0.112	0.584 ± 0.082	0.591 ± 0.049	0.017 ± 0.086
Clade II	8	160	5.175 ± 1.788	3.168 ± 0.951	1.147 ± 0.353	0.524 ± 0.119	0.565 ± 0.132	0.072 ± 0.089
Clade III	6	120	4.531 ± 0.421	2.611 ± 0.257	0.751 ± 0.090	0.372 ± 0.062	0.403 ± 0.048	0.109 ± 0.083
Clade IV	8	160	4.150 ± 0.578	2.300 ± 0.309	0.863 ± 0.122	0.412 ± 0.074	0.437 ± 0.059	0.086 ± 0.116
Clade V	13	260	3.969 ± 0.706	2.288 ± 0.287	0.849 ± 0.160	0.438 ± 0.086	0.440 ± 0.072	0.021 ± 0.106

Among the six evolutionary scenarios evaluated in ABC modeling, we estimated the historical gene flow between each sister clades of model 4. Significant levels of gene flow (2*Nm* = 4.39–5.28) were detected among five clades (Figure 3D). Particularly, sister clades sharing a phylogenetic divergence relationship were identified as main contributors to gene flow in *P. sikkimensis* . In contrast, clades II and V, which are geographically adjacent but lack a direct divergence relationship, exhibited substantial bidirectional gene flow, with values of 4.95 (II–V) and 4.43 (V–II) (Figure 3D).

### 3.5 Species Distribution Modeling

**FIGURE 4:**
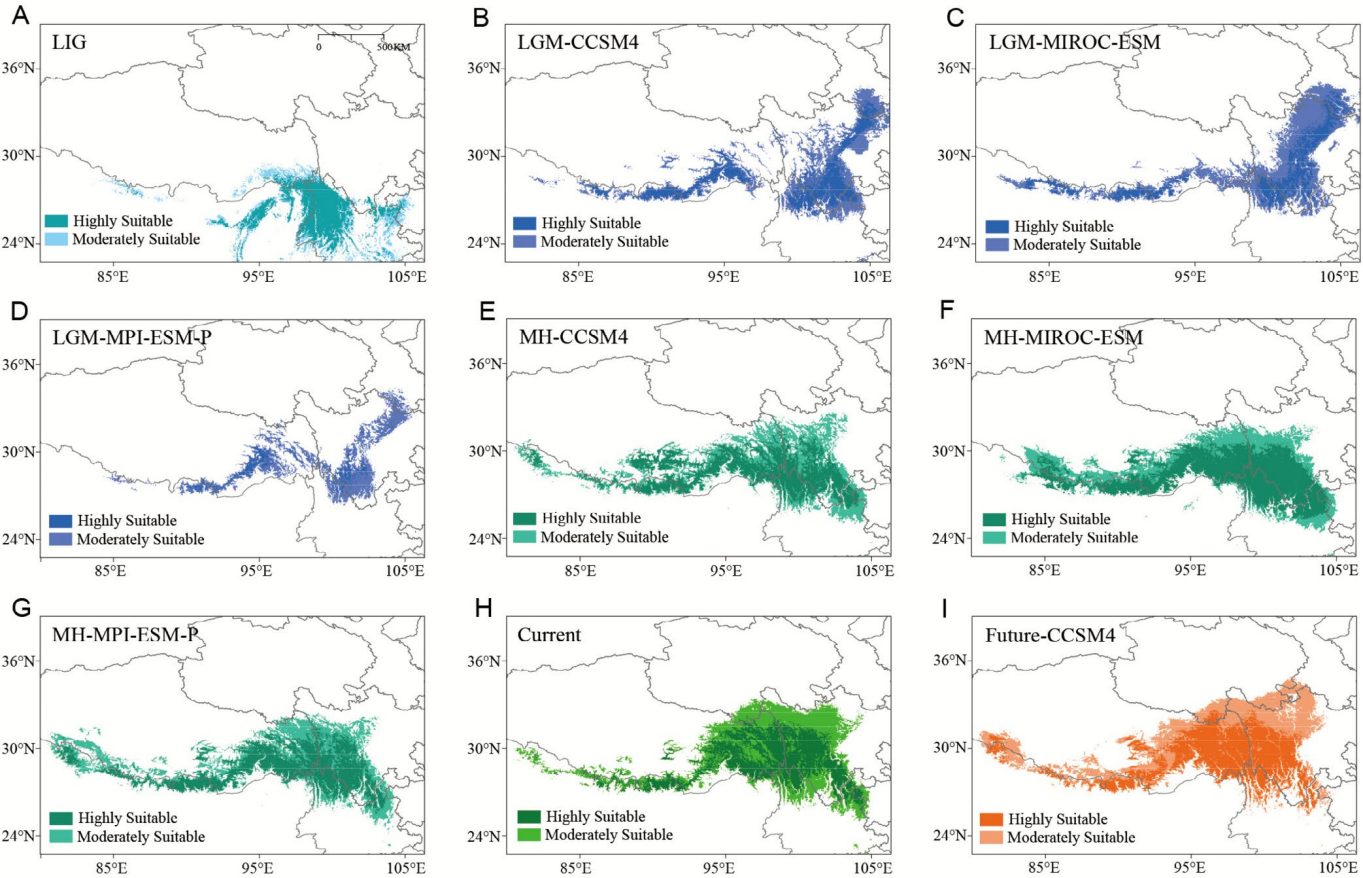
Predicted distribution of *Primula sikkimensis* based on ecological niche modeling using Maxent: (A) Last Interglacial (LIG; 0.12–0.14 Ma), (B–D) Last Glacial Maximum (LGM; ~0.022 Ma) simulated by CCSM4, MIROC‐ESM, and MPI‐ESM‐P respectively, (E–G) Mid‐Holocene (MH; ~0.006 Ma) simulated by CCSM4, MIROC‐ESM, and MPI‐ESM‐P respectively, (H) Present (1950–2000), and (I) Future (~2050) climate conditions.

Current distribution predictions were generally good representations of the actual distributions of the species, the only exception being the predicted occurrence of populations in the west Himalaya and south Sichuan Basin, where the species does not occur at present (Figure 4H). Palaeodistribution modeling indicated a trend of southward movement during the LIG, but with a comparable distribution range compared to the present (Figure 4A). The predictions from CCSM4, MIROC‐ESM, and MPI‐ESM‐P for MH conditions yielded continuous and similar patterns of distribution compared to current conditions, with MIROC‐ESM showing a slightly larger distribution, particularly in highly suitable areas (Figure 4E–G). During the LGM, all three Palaeodistribution models suggested an expansion of suitable habitat compared with the predictions for the present and MH, primarily due to colonization in the northeast (Figure 4B–D).

## 4 Discussion

Phylogeographic investigations of alpine flora have revealed two contrasting biogeographic scenarios: postglacial recolonization of the inner QTP platform from LGM refugia located in lower‐elevation zones along the eastern and southeastern plateau margins, alongside emerging evidence for persistent microrefugia on the plateau proper that may have sustained populations through the LGM and potentially earlier Pleistocene glacial maxima (Wang et al. 2009, 2019; Opgenoorth et al. 2010). Divergent phylogeographic trajectories among alpine lineages are evidenced by contrasting recolonization routes and significantly varying estimates of postglacial expansion timing, with notable discrepancies observed between herbaceous taxa and woody shrubs. As quintessential QTP *sl* representatives, *Primula*, *Rhododendron*, *Pedicularis*, and *Gentiana* collectively embody the region's extraordinary species richness and unparalleled endemism. Comparative phylogeographic investigations across these hyperdiverse clades, particularly integrating their distinct mating and dispersal strategies, would elucidate how heterogeneous landscape evolution and biotic interactions have jointly shaped the QTP's spectacular radiation patterns.

Phylogeographic studies of plant species across QTP *sl* and its surrounding regions indicated that some species likely persisted in geographically isolated refugia during the LGM (Liu et al. 2012; Wen et al. 2014). The Himalayan region has been recognized as one of the important refugia within the peripheral zones of the QTP *sl* (Wang et al. 2010, 2019; Jia et al. 2011; Ren et al. 2017). For example, Ren et al. (2017) identified three potential refugia for *P. tibetica* , a congeneric alpine species of *P. sikkimensis* sharing similar elevational ranges (2600–5000 m), located in the eastern, central and southwestern Himalayas. Meanwhile, the Hengduan Mountains region was more widely acknowledged as critical refugia not only for *Primula* species (e.g., *P. fasciculate* and *P. nutans* , Ren et al. 2018) but also other symbol species‐rich genera of the QTP *sl*, such as *Rhododendron* (Ericaceae), *Pedicularis* (Orobanchaceae) and *Gentiana* (Gentianaceae) (Favre et al. 2016; Li et al. 2016; Sun et al. 2017; Wang et al. 2022). Using combined molecular markers, we identified a key ancestral lineage (clade III) in the western Hengduan Mountains region that served as the source for subsequent sequential expansions leading to the origin of QTP *ss* lineages (Figures 1, 3). Another ancestral lineage comprised clades from the core Hengduan Mountains region (clade I) and eastern Himalayas (clade II), which showed relatively low genetic divergence. No clear evidence suggests that these two clades derived from one another, while the persistence of ancient haplotypes in each region may imply that they served as independent refugia for the species. Furthermore, the low level of differentiation between the two clades likely results from recent gene flow, possibly during the LGM, as supported by multiple studies indicating the co‐occurrence of refugia in the Himalaya and the core Hengduan mountains region (Ren et al. 2017; Wang et al. 2019). These results, as well as the projected habitat at the LGM (Figure 4B–D), suggested that both the Hengduan Mountains region and eastern Himalaya likely served as refugia for *P. sikkimensis* during the LGM, enabling species survival through adverse climate oscillations while facilitating the accumulation of genetic diversity for subsequent expansion. Although a growing body of evidence supports the existence of persistent microrefugia on the plateau interior that maintained populations during the LGM and possibly earlier Pleistocene glacial maxima (Chen et al. 2014; Wang et al. 2014, 2019; Lu et al. 2015), there is currently no indication that *Primula* species, including *P. sikkimensis* , shared a similar history.

In the present study, range‐wide genetic variation of *Primula sikkimensis* was surveyed using markers of both maternally inherited whole chloroplast genomes and biparental nuclear genomes. We found that the QTP *ss* populations were clustered in two groups (clades IV and V) and were sequentially derived from the northern marginal clade (III) of HHM lineage (Figures 1, 2). The chloroplast genome haplotype tree, together with lineage differentiation modeling, strongly suggest that the Hengduan Mountains region provided refugia for *P. sikkimensis* during the Quaternary glaciations, and that a series of founder effects occurred during the recolonization of this species into the QTP *ss* region. This inference is consistent with the findings in genetic diversity analysis of both plastid and nuclear genes, such as the much lower allele diversity in the QTP *ss* than in the Hengduan Mountains region, and the gradual decrease in nucleotide diversity of plastid genes from the core region of the Hengduan Mountains to the northwest of the Hengduan Mountains and finally to QTP *ss*. Furthermore, the genetic load measured by the ratio of nonsynonymous to synonymous mutations (*π*_N_:*π*_S_ or *θ*_N_:*θ*_S_) was gradually increased from the Hengduan Mountains lineage to the QTP *ss* lineage. This pattern is a recognized signature of a recent demographic history of bottleneck, or of a drastic mating system transition from the ancestral distyly (obligate outcrossing) to homostyly (where stigma and anthers are at same height, promoting self‐fertilization), as revealed in our previous study (Wang et al. 2021). However, the field investigation indicated that the QTP *sl* populations all presented floral dimorphism and did not involve the shift of the mating system from outcrossing to selfing as reported in other *Primula* species, e.g., *P. chungensis* (Zhou et al. 2017), *P. wannanensis* (Zhang et al. 2021), and *P. oreodoxa* (Zeng et al. 2024). Therefore, the higher genetic load in QTP *ss* populations should be another line of evidence for the derived status relative to the Hengduan Mountains populations.

When a species experiences a series of founder events, both allelic diversity and expected heterozygosity tend to decline significantly as the number of founding episodes between populations increases (Clegg et al. 2002; Pruett and Winker 2005). Unexpectedly, peripheral clade V maintained similar genetic diversity to clade IV while showing slightly reduced genetic load (Tables 1, 2). The rebound of genetic diversity in clade V may have been caused by following factors. First, secondary contact and gene exchange of isolated populations between clades II and V. Our demographic analyses indicated that both lineages experienced recent population expansion during the late Pleistocene. Furthermore, gene flow analyses revealed substantial bidirectional migration between these clades (Figure 3E), which is corroborated by shared chloroplast haplotypes in the phylogenetic network (Figure 2). Previous phylogeographic studies have shown that most plant species on the QTP *Sl* recolonized the central platform from their LGM refugia located along the eastern or southeastern margins (i.e., Hengduan Mountains region), whereas northward recolonization directly from the Himalaya appears unlikely (Yu et al. 2017; Wang et al. 2019). However, the genetic admixture between clades II and V of *P. sikkimensis* suggests that postglacial population contact between the QTP *Ss* and the Himalaya was likely feasible particularly in eastern Himalayan region. Another possible explanation for the high genetic diversity in peripheral populations (clade V) might be linked to the widespread occurrence of interspecific hybridization and/or introgression in *Primula*, with several species even being considered of hybrid origin (Guggisberg et al. 2008; Mora‐Carrera et al. 2025). In the QTP *ss* region, there are potential hybridization partners with *P. sikkimensis* , particularly *P. alpicola* and *P. florindae*, whose distribution ranges overlapped with clade V populations. Although further evidence is required, this inference was supported by the haplotype tree, in which haplotypes from clade V occupy the basal phylogenetic positions (Figure 2B). Collectively, these findings suggest that post‐LGM colonizers expanding across the QTP plateau may not necessarily exhibit reduced genetic diversity, as secondary contact dynamics and interspecific hybridization following Pleistocene glacial retreat could generate elevated diversity reservoirs (Guggisberg et al. 2006; Stubbs et al. 2024). This enhanced genomic complexity may facilitate adaptation to environmental changes, particularly for species such as *Primula* that inhabit Alpine and Arctic regions (Guggisberg et al. 2009). Importantly, such mechanisms caution against overinterpreting high genetic diversity or haplotype variation as unambiguous evidence for plateau‐based glacial refugia in other alpine species, unless corroborated by explicit tests of introgression and hybridization signatures.

Our use of complete chloroplast genomes (cpDNA), rather than traditional short cpDNA fragments, provided critical advantages but also introduced limitations for reconstructing the phylogeographic history of *P. sikkimensis* . First, the substantially increased density of informative sites (> 100× more variable loci than typical fragment analyses) enabled high‐resolution lineage delimitation. This was essential for detecting subtle founder effects during QTP recolonization, such as tracing the sequential derivation of clades IV and V from clade III. Second, inclusion of both coding and non‐coding regions captured complementary evolutionary signals; non‐coding loci refined haplotype networks (e.g., shared haplotypes indicating clade II/V gene flow), while coding regions permitted, third, and most significantly, direct estimation of genetic load through nonsynonymous/synonymous mutation ratios (*π*_N_/*π*_S_). This proved indispensable for inferring recent bottlenecks in derived QTP *ss* lineages without mating‐system shifts, an insight that could not be attained through fragment‐based or frequency‐focused approaches. However, two constraints merit consideration: While multiplexed sequencing mitigated costs, whole‐cp genome assembly still necessitated reduced within‐population sampling (compared to fragment‐based studies) due to per‐sample sequencing expenses. This potentially obscured fine‐scale haplotype frequency patterns across the plateau periphery. Additionally, the exceptional resolving power of cpDNA genomes often yields population‐specific haplotypes, complicating traditional analyses reliant on shared haplotype distributions (e.g., spatial clustering of high‐frequency haplotypes as refugial indicators). Consequently, while we robustly reconstructed colonization routes and refugial origins, our approach was less suited for demographic inferences dependent on haplotype frequency spectra. Future studies targeting frequency‐based metrics could strategically combine high‐throughput nuclear SNPs with subsetted cpDNA genomes to balance resolution and analytical breadth.

## 5 Conclusions

Our findings further indicate that *Primula sikkimensis*, an alpine‐distributed primrose species, maintained separate glacial refugia in the Hengduan Mountains and eastern Himalayas during the LGM. Postglacial range expansions onto heartland of the QTP plateau were accompanied by gene flow arising from both intraspecific secondary contact between previously isolated populations and interspecific hybridization events, which collectively enhanced genetic diversity and adaptive capacity in plateau‐interior populations. Future investigations employing reduced‐representation sequencing (i.e., RAD‐seq) (Ren et al. 2017), population transcriptomics (Zeng et al. 2024), or whole‐genome resequencing integrated with chromosome‐level assemblies (Stubbs et al. 2024) could substantially enhance our understanding of historical demographic fluctuations and cryptic interspecific introgression events in this system. Furthermore, comparative phylogeographic analyses across multiple congeneric species would provide critical insights into the spatiotemporal distribution patterns of alpine taxa across the QTP *sl* and their evolutionary consequences for speciation and diversification processes.

## Author Contributions

**Wei Zhou:** conceptualization (equal), formal analysis (equal), investigation (equal), writing – review and editing (equal). **Hua‐Ying Sun:** investigation (equal), writing – original draft (equal). **Yu‐Ting He:** investigation (equal). **Zhi‐Hua Zeng:** formal analysis (equal), investigation (equal). **Yuan‐Mi Wu:** formal analysis (equal), investigation (equal). **Li Zhong:** investigation (equal). **Qing‐Hong Feng:** investigation (equal). **Hui‐Ying Gong:** investigation (equal). **Xin Wang:** investigation (equal). **Hong Wang:** investigation (equal). **Zhi‐Kun Wu:** conceptualization (equal), investigation (equal).

## Funding

This work was supported by the National Natural Science Foundation of China, 32470394.

## Conflicts of Interest

The authors declare no conflicts of interest.

## Supporting information


**Figure S1:** Models that were tested for lineage differentiation history of the *Primula sikkimensis* using Approximate Bayesian Computation (ABC) modeling. Ts represent the recent divergence time for most closed lineages in generations ago. Ns are the effective population size of lineage or the common ancestor of the lineages.
**Figure S2:** Determination of the optimal number of genetic clusters (*K*) for 48 populations of *Primula sikkimensis* using the Δ*K* method of Evanno et al. (2005). The plot shows the mean Δ*K* value (rate of change in the log probability of data) over 20 independent runs for each value of *K*, ranging from 2 to 8. The peak at *K* = 5 indicates the most likely number of genetically distinct groups within the dataset, based on 10 microsatellite loci.
**Figure S3:** Diagram of four demographic history models for *Primula sikkimensis*, depicting changes in population size (horizontal axis) over time (vertical axis). Time increases upward, with T3 (oldest), T2, T1, and the present (0) labeled. Population sizes include ancestral size (NA), expanded size (Nb), bottleneck size (Nc), and present size (N1).
**Table S1:** Population sampling information of *Primula sikkimensis*.
**Table S2:** Posterior probabilities of modeled scenarios obtained by logistic regression of 1% of the closest simulated datasets for levels of species and clade.
**Table S3:** Estimations of posterior distributions of parameters for the best fitting model (model 4) of demographic history of *Primula sikkimensis*.

**Table S4:** Parameters of the genetic diversity estimated from microsatellite genotypes for each population of *Primula sikkimensis*.

## Data Availability

Plastid genome assembles used in this study have been deposited in the GenBank database with accession numbers provided in Table S1.
