# Supplementary material for: Multiple Glacial Refugia and Complex Postglacial Dynamics of Primula sikkimensis (Primuaceae) in the Heterogeneous Qinghai‐Tibet Plateau
Source: Ecol Evol. 2026 Feb 3;16(2):e73024. doi: 10.1002/ece3.73024 (PMC12865663; doi:10.1002/ece3.73024)
Supplement: Supplementary file 1 — Figure S1: Models that were tested for lineage differentiation history of the Primula sikkimensis using Approximate Bayesian Computation (ABC) modeling. Ts represent the recent divergence time for most closed lineages in generations ago. Ns are the effective population size of lineage or the common ancestor of the lineages. Figure S2: Determination of the optimal number of genetic clusters (K) for 48 populations of Primula sikkimensis using the ΔK method of Evanno et al. (2005). The plot shows the mean ΔK value (rate of change in the log probability of data) over 20 independent runs for each value of K, ranging from 2 to 8. The peak at K = 5 indicates the most likely number of genetically distinct groups within the dataset, based on 10 microsatellite loci. Figure S3: Diagram of four demographic history models for Primula sikkimensis, depicting changes in population size (horizontal axis) over time (vertical axis). Time increases upward, with T3 (oldest), T2, T1, and the present (0) labeled. Population sizes include ancestral size (NA), expanded size (Nb), bottleneck size (Nc), and present size (N1). Table S1: Population sampling information of Primula sikkimensis. Table S2: Posterior probabilities of modeled scenarios obtained by logistic regression of 1% of the closest simulated datasets for levels of species and clade. Table S3: Estimations of posterior distributions of parameters for the best fitting model (model 4) of demographic history of Primula sikkimensis. [file ECE3-16-e73024-s002.docx]

## Supporting information for

**Multiple glacial refugia and complex postglacial dynamics of *Primula sikkimensis* (Primuaceae) in the heterogeneous Qinghai-Tibet Plateau**

Hua-Ying Sun^1, 2†^, Yu-Ting He^2, 3†^, Zhi-Hua Zeng^2, 4†^, Yuan-Mi Wu^2, 4†^, Li Zhong^5^, Qing-Hong Feng^6^, Hui-Ying Gong^2, 4^, Xin Wang^2, 4^, Hong Wang^2^, Zhi-Kun Wu^7^* and Wei Zhou^2, 8^*

## Supporting information


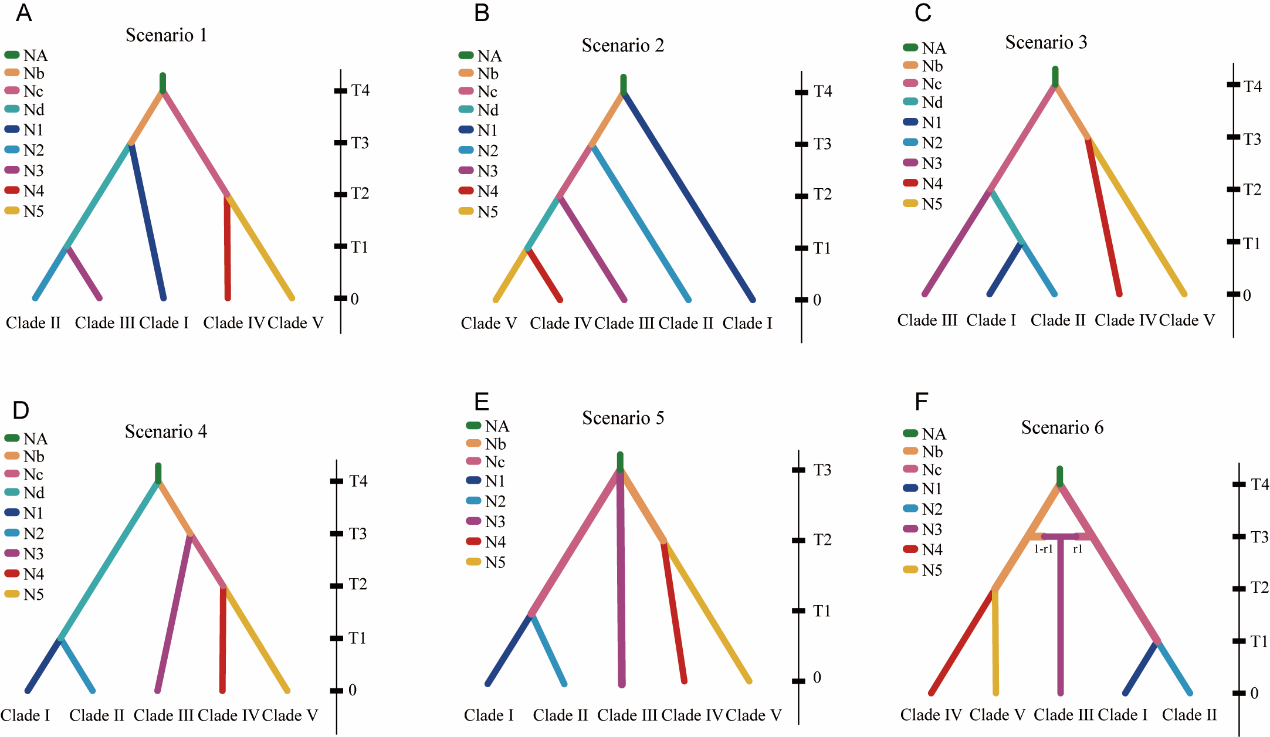


**Figure S1** Models that were tested for lineage differentiation history of the *Primula sikkimensis* using Approximate Bayesian Computation (ABC) modelling. Ts represent the recent divergence time for most closed lineages in generations ago. Ns are the effective population size of lineage or the common ancestor of the lineages.


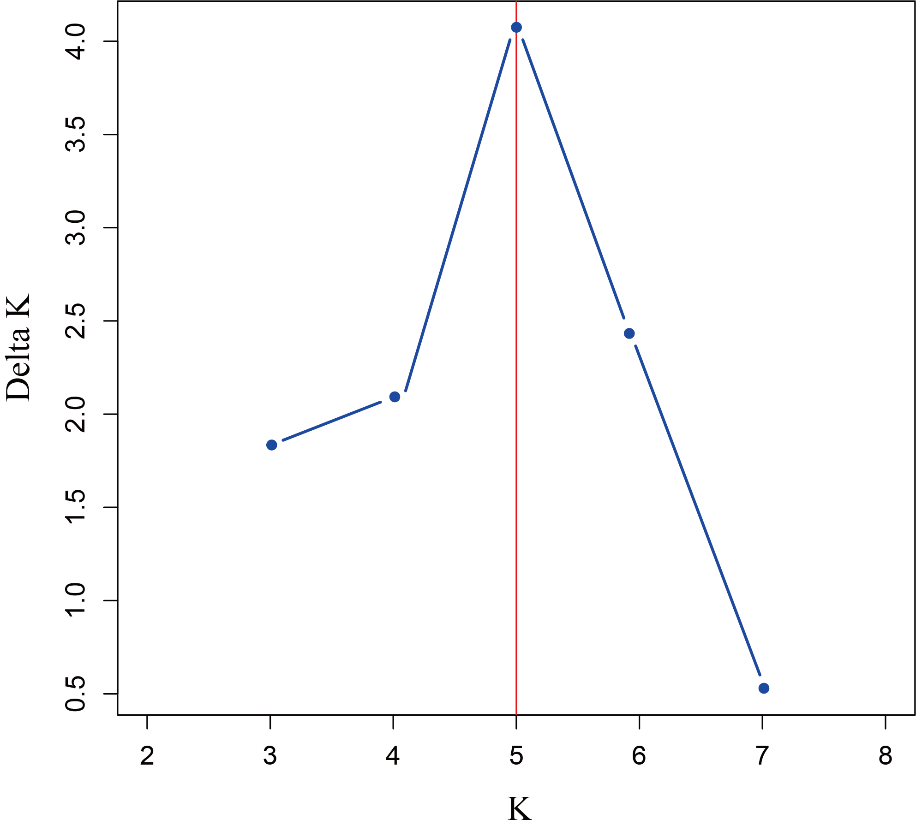


**Figure S2** Determination of the optimal number of genetic clusters (*K*) for 48 populations of *Primula* *sikkimensis* using the Δ*K* method of Evanno et al. (2005). The plot shows the mean Δ*K* value (rate of change in the log probability of data) over 20 independent runs for each value of *K*, ranging from 2 to 8. The peak at *K* = 5 indicates the most likely number of genetically distinct groups within the dataset, based on ten microsatellite loci.


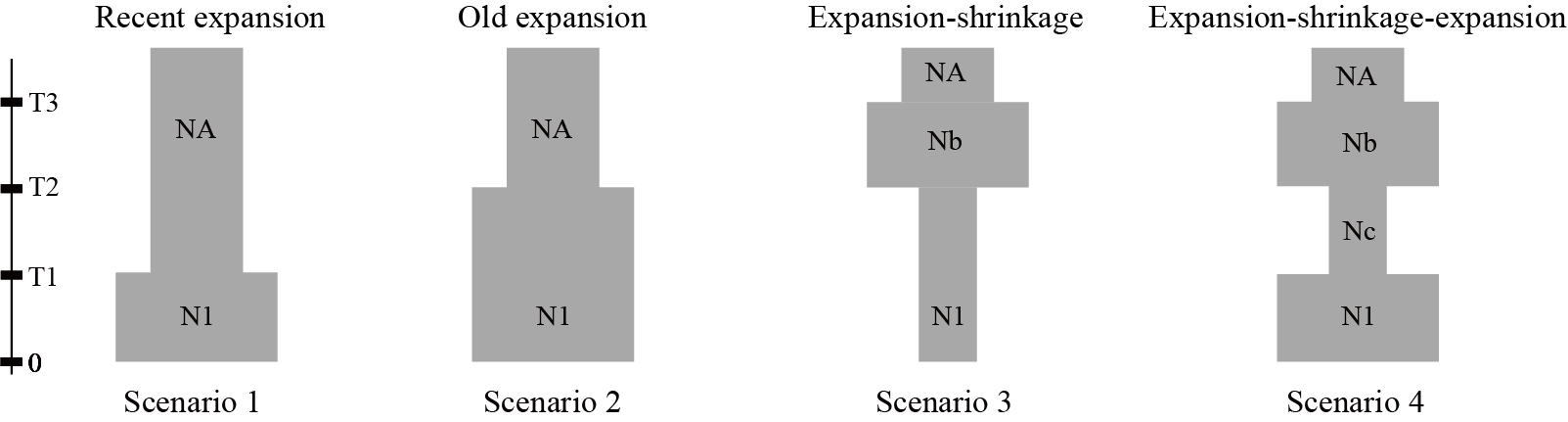


**Figure S3** Diagram of four demographic history models for *Primula sikkimensis*, depicting changes in population size (horizontal axis) over time (vertical axis). Time increases upward, with T3 (oldest), T2, T1, and the present (0) labeled. Population sizes include ancestral size (NA), expanded size (Nb), bottleneck size (Nc), and present size (N1).

**Table S1** population sampling information of *Primula sikkimensis*.

| Code | Sample Number | Location | Latitude (N) | Longitude  (E) | Altitude (m) | GenBank accession |
| --- | --- | --- | --- | --- | --- | --- |
| SGA | 20 | Yunnan | 27.57 | 99.85 | 3359 | PX068063 |
| SGB | 20 | Yunnan | 28.38 | 99.01 | 4273 | PX068064 |
| CDX | 20 | Tibet | 31.70 | 94.98 | 4091 | PX068080 |
| NQA | 20 | Tibet | 31.89 | 94.18 | 4276 | PX068070 |
| LZA | 20 | Tibet | 29.62 | 94.67 | 4427 | PX068076 |
| LZB | 20 | Tibet | 29.77 | 95.70 | 3729 | PX068075 |
| LZC | 20 | Tibet | 29.66 | 95.33 | 3846 | PX068074 |
| ZDA | 20 | Yunnan | 27.80 | 99.59 | 4370 | PX068061 |
| ZDB | 20 | Yunnan | 28.13 | 99.89 | 4060 | PX068060 |
| ZDC | 20 | Yunnan | 28.16 | 99.91 | 4397 | PX068059 |
| DCA | 20 | Sichuan | 28.93 | 100.29 | 4060 | PX068056 |
| XCA | 20 | Sichuan | 29.02 | 99.72 | 3761 | PX068054 |
| DQA | 18 | Yunnan | 28.39 | 98.99 | 4198 | PX068065 |
| CDY | 20 | Tibet | 29.23 | 98.68 | 3865 | PX068079 |
| CDZ | 20 | Tibet | 29.28 | 98.68 | 3863 | PX068078 |
| JCD | 20 | Tibet | 29.55 | 98.21 | 3607 | PX068077 |
| CDA | 20 | Tibet | 29.62 | 98.15 | 4158 | PX068053 |
| CDB | 20 | Tibet | 29.69 | 97.94 | 4139 | PX068052 |
| CDC | 20 | Tibet | 30.42 | 97.21 | 4223 | PX068051 |
| CDD | 20 | Tibet | 30.81 | 96.69 | 4452 | PX068050 |
| CDE | 20 | Tibet | 30.86 | 96.55 | 4693 | PX068049 |
| CDF | 20 | Tibet | 30.87 | 96.54 | 4363 | PX068048 |
| CDG | 20 | Tibet | 30.85 | 96.18 | 3429 | PX068047 |
| CDI | 20 | Tibet | 31.09 | 96.99 | 4024 | PX068046 |
| CDJ | 20 | Tibet | 31.08 | 96.95 | 4583 | PX068045 |
| CDK | 20 | Tibet | 31.11 | 96.88 | 4017 | PX068044 |
| CDL | 20 | Tibet | 30.86 | 95.45 | 3624 | PX068043 |
| CDM | 20 | Tibet | 30.81 | 95.21 | 4528 | PX068042 |
| CDN | 20 | Tibet | 30.79 | 95.02 | 4085 | PX068041 |
| CDO | 20 | Tibet | 30.99 | 94.66 | 3898 | PX068040 |
| CDP | 20 | Tibet | 31.02 | 94.58 | 4698 | PX068039 |
| CDQ | 20 | Tibet | 30.43 | 93.75 | 4127 | PX068038 |
| CDR | 20 | Tibet | 31.14 | 96.48 | 4162 | PX068037 |
| CDS | 20 | Tibet | 31.70 | 94.96 | 4390 | PX068036 |
| CDT | 20 | Tibet | 31.67 | 94.84 | 4298 | PX068035 |
| SNA | 20 | Tibet | 29.04 | 92.35 | 4892 | PX068069 |
| SNB | 20 | Tibet | 29.05 | 92.40 | 3693 | PX068068 |
| SNC | 20 | Tibet | 29.47 | 92.73 | 4960 | PX068067 |
| SND | 20 | Tibet | 29.39 | 92.75 | 4394 | PX068066 |
| LZD | 20 | Tibet | 28.77 | 93.46 | 4066 | PX068073 |
| LZE | 20 | Tibet | 29.34 | 97.06 | 4388 | PX068072 |
| LZF | 20 | Tibet | 29.74 | 97.21 | 3106 | PX068071 |
| ZDD | 20 | Yunnan | 27.78 | 99.58 | 4011 | PX068058 |
| SGC | 20 | Yunnan | 27.78 | 99.59 | 3837 | PX068062 |
| CDU | 20 | Tibet | 28.41 | 98.99 | 3331 | PX068034 |
| CDV | 20 | Tibet | 29.59 | 98.30 | 4457 | PX068081 |
| CDW | 20 | Tibet | 29.67 | 98.13 | 3917 | PX068033 |
| DCB | 20 | Sichuan | 28.90 | 100.28 | 4418 | PX068055 |

**Table S2** Posterior probabilities of modeled scenarios obtained by logistic regression of 1% of the closest simulated datasets for levels of species and clade.

|  | Scenario 1 | Scenario 2 | Scenario 3 | Scenario 4 | Scenario 5 | Scenario 6 |
| --- | --- | --- | --- | --- | --- | --- |
| species | 0.00277 (0.00222, 0.00332) | 0.03576 (0.01404, 0.05748) | 0 (0, 0) | 0.88675 (0.85524, 0.91826) | 0.06545 (0.04362, 0.08728) | 0.00053 (0.00041, 0.00065) |
| Clade I | 0.2337 (0.2283, 0.2391) | 0.2437 (0.2333, 0.2540) | 0.1910 (0.1843, 0.1977) | 0.3243 (0.3180, 0.3307) | - | - |
| Clade II | 0.2132 (0.2055, 0.2210) | 0.2405 (0.2252, 0.2558) | 0.2020 (0.1940, 0.2100) | 0.3429 (0.3235, 0.3623) | - | - |
| Clade III | 0.2984 (0.2609, 0.3360) | 0.1127 (0.1060, 0.1194) | 0.3346 (0.3139, 0.3553) | 0.2546 (0.2444, 0.2648) | - | - |
| Clade IV | 0.2889 (0.2752, 0.3026) | 0.2867 (0.2703, 0.3031) | 0.0144 (0.0108, 0.0180) | 0.4108 (0.3792, 0.4424) | - | - |
| Clade V | 0.2220 (0.1965, 0.2475) | 0.3001 (0.2696, 0.3306) | 0.0220 (0.0168, 0.0272) | 0.4484 (0.4433, 0.4535) | - | - |

**Table S3** Estimations of posterior distributions of parameters for the best fitting model (model 4) of demographic history of *Primula sikkimensis*.

| Parameter | mean | median | mode | 95% CI | |
| --- | --- | --- | --- | --- | --- |
| N1 | 6.70E+05 | 6.76E+05 | 6.47E+05 | 3.59E+05 | 9.52E+05 |
| N2 | 6.68E+05 | 6.75E+05 | 6.73E+05 | 3.47E+05 | 9.54E+05 |
| N3 | 2.26E+05 | 1.73E+05 | 1.30E+05 | 7.56E+04 | 5.96E+05 |
| N4 | 3.28E+05 | 2.75E+05 | 2.17E+05 | 1.20E+05 | 7.46E+05 |
| N5 | 6.17E+05 | 6.05E+05 | 5.43E+05 | 3.57E+05 | 9.22E+05 |
| T1 | 4.43E+04 | 4.36E+04 | 3.78E+04 | 9.74E+03 | 8.12E+04 |
| Nd | 7.04E+05 | 7.24E+05 | 7.78E+05 | 3.54E+05 | 9.68E+05 |
| T2 | 7.38E+04 | 7.68E+04 | 8.68E+04 | 3.93E+04 | 9.74E+04 |
| Nc | 4.21E+05 | 3.75E+05 | 1.69E+05 | 6.82E+04 | 9.08E+05 |
| T3 | 9.85E+04 | 9.98E+04 | 1.03E+05 | 4.90E+04 | 1.43E+05 |
| Nb | 1.48E+05 | 7.32E+04 | 1.33E+04 | 8.07E+03 | 5.84E+05 |
| T4 | 1.76E+05 | 1.80E+05 | 1.98E+05 | 1.41E+05 | 1.98E+05 |
| NA | 7.40E+04 | 8.04E+04 | 9.95E+04 | 2.65E+04 | 9.85E+04 |
| *μ* | 8.54E-06 | 8.92E-06 | 1.00E-05 | 5.71E-06 | 1.00E-05 |
| *P* | 2.74E-01 | 2.85E-01 | 3.00E-01 | 2.05E-01 | 3.00E-01 |

**Table S4** Parameters of the genetic diversity estimated from microsatellite genotypes for each population of *Primula sikkimensis*.

See a separate Excel document.
